# Chemotherapy-induced liver injury in metastatic colorectal cancer: about 48 cases

**DOI:** 10.11604/pamj.2018.30.198.15548

**Published:** 2018-07-05

**Authors:** Faten Limaiem, Saadia Bouraoui

**Affiliations:** 1 Department of Pathology, University of Tunis El Manar, Tunis Faculty of Medicine, 1007, Tunisia

**Keywords:** Colorectal cancer, metastasis, chemotherapy, hepatotoxicity

## Abstract

Neoadjuvant chemotherapy of colorectal liver metastases can induce hepatotoxicity in noncancerous liver. The aim of the present study was to describe the chemotherapy-induced major changes in the hepatic parenchyma and their prognostic impact. We undertook a retrospective study of 48 cases of colorectal liver metastases treated with neoadjuvant therapy followed by liver resection. These cases were collected at the Pathology Department of Mongi Slim Hospital over a 2-year period (July 2015-February 2018). Our series consisted of 27 men and 21 women with a sex-ratio (M/F = 1.28). The average age of our patients was 57.68 years old with extremes ranging from 30 to 75 years old. All patients received chemotherapy with FOLFOX. From a total of 48 operative specimens examined, we found 24 cases (50%) of non-systematized steatosis, grade 1 sinusoidal obstruction syndrome (n = 12) and grade 2 sinusoidal obstruction syndrome (n = 12), regenerative nodular hyperplasia (n = 3), portal and/or lobular inflammatory infiltrate (n = 6). In three cases, no abnormalities were reported in the liver parenchyma. Surgical margins were < 1 mm in seven cases and were invaded in four cases. Preoperative chemotherapy is associated with regimen-specific liver injury. The presence of such an injury may have a negative impact on the functional reserve of the liver, thereby increasing the risk of surgical morbidity and mortality.

## Introduction

Liver metastasis is the most common complication of colorectal cancer and approximately 50% of patients develop colorectal liver metastases (CLM) during the course of their disease [1, 2]. It is universally accepted that patients with inoperable disease should be treated, where possible, with aggressive chemotherapy with a view to downstaging disease such that curative surgery can be offered [3]. Preoperative chemotherapy is associated with regimen-specific liver injury. The presence of such an injury may have a negative impact on the functional reserve of the liver, thereby increasing the risk of surgical morbidity and mortality. In this paper, we report our experience with liver metastases over the past three years. Our aim was to describe the chemotherapy-induced major changes in the hepatic parenchyma and their prognostic impact.

## Methods

**Patient selection criteria:** This was a retrospective analysis of data collected from patients with CLM managed in our University Hospital Mongi Slim La Marsa, from July 2015 to February 2018. Eighty-four patients met the inclusion criteria which were the following: hepatectomy for documented CLM, no underlying chronic liver disease (nonalcoholic, hepatitis B or C virus, or autoimmune chronic liver disease or genetic haemochromatosis) and with sufficient non-tumorous liver parenchyma for pathologic analysis. All patients received preoperative systemic chemotherapy.

**Preoperative evaluation**: All patients underwent a preoperative evaluation including an abdominal and thoracic CT scan. Patients were considered for hepatectomy if all detected tumors could be removed completely with grossly negative surgical margins and a safe liver remnant volume.

**Indication and regimens of systemic chemotherapy**: In resectable patients, indication for preoperative chemotherapy was to downsize the tumors preoperatively in view of a function-sparing resection or to ensure negative margins and to assess tumor's response to chemotherapy. The patients with nonresectable CLM at presentation received “induction” chemotherapy which aimed at downsizing the CLM to switch the patients from a “non-resectable” status to a “resectable” status. Systemic chemotherapy was administered before hepatic surgery in all cases. The chemotherapeutic agents used were 5-FU, 5-FU with leucovorin and oxaliplatin (FOLFOX).

**Pathologic analysis**: All slides, which were originally prepared from formalin-fixed and paraffin-embedded tissues, were reviewed. Representative slides of non-tumorous hepatic tissue located as far as possible from the tumor were selected for the study. The morphological analyses were performed using slides stained with hematoxylin and eosin and Masson trichrome stain. The slides were examined by a single pathologist with hepatobiliary expertise. Hepatic steatosis was classified into 3 grades: less than or equal to 30%, between 30% and 60% and greater than 60%. Steatohepatitis was evaluated according to the semiquantitative score of Kleiner et al and the NASH activity score (NAS) obtained by the addition of the steatosis (0 ≤ 5%, 1 = 5-33%, 2 = 33-66% and 3 ≥ 66%), lobular inflammation (0 = no site, 1 ≤ 2 sites, 2 = two to four sites and 3 ≥ four sites per ×200 field) and hepatocyte ballooning (0 = absent, 1 = several ballooned hepatocytes, 2 = numerous ballooned hepatocytes, or predominant hepatocyte ballooning) scores. A NAS score ≥ 5 was in favor of steatohepatitis, and a score less than 3 excluded steatohepatitis. Sections were examined for the presence of vascular lesions such as sinusoidal dilatation classified into grade I (minimal centrilobular dilatation), II (dilatation occupying 2/3 of the lobule) and III (dilatation occupying all of the lobule). Portal fibrosis was estimated according to the Metavir score: absent (F0), portal fibrosis without septa (F1), portal fibrosis with several septa (F2), numerous septa without cirrhosis (F3), and cirrhosis (F4). Finally, lesions secondary to intraoperative manipulation of the surgical specimen were defined by periportal or centrilobular hepatocyte necrosis with polymorphonuclear neutrophile infiltrate and were called “surgical hepatitis”.

**Studied criteria**: Demographic data (age, gender), pathological variables (number and size of CLM, pTNM stage), chemotherapy characteristics (type of chemotherapy, number of courses, dose and duration of chemotherapy and interval between the end of chemotherapy and liver surgery), surgical modalities and postoperative outcomes (mortality, morbidity and length of stay) were recorded.

## Results

**Figure 1:**
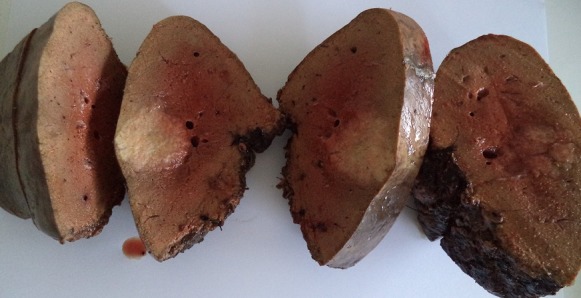
Macroscopic findings of colorectal liver metastasis; cut section of the liver, showing a well-delineated sub-capsular whitish tumor

**Figure 2:**
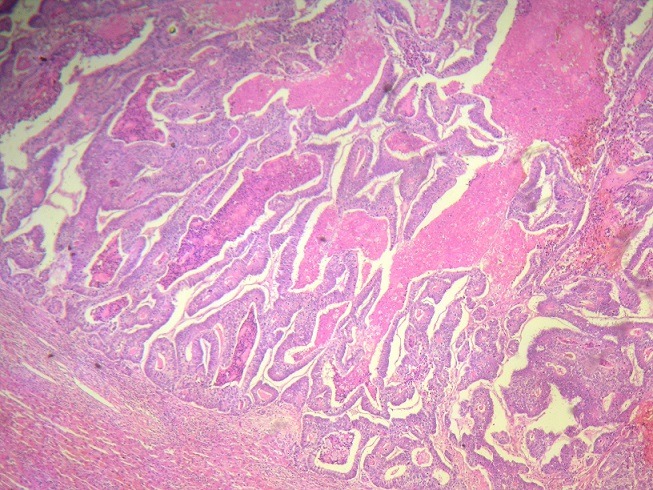
Histological findings of colorectal liver metastasis. Partial pathologic response with necrosis; pathologic response evaluation of colorectal liver metastases according to Rubbia-Brandt is classified as TRG4 and is evaluated as a minor response (more than 50% of residual tumor cells) according to Blazer classification (hematoxylin and eosin, magnification × 200)

**Patients**: From July 2015 to February 2018, a total of 58 patients underwent hepatectomy for CLM in our department. Among these 58 patients, 48 (82,75%) received neoadjuvant chemotherapy. Fourty-eight patients satisfying the inclusion criteria were analyzed. There were 27 males and 21 females with a mean age of 57,68 years (range: 30-75 years). Colorectal liver metastasis was synchronous in 10 patients (20,83%). The mean number of metastases was 2 (range: 1-15) (Figure 1,Figure 2) with a mean diameter of 2.23cm (range: 0.4-10 cm).

**Figure 3:**
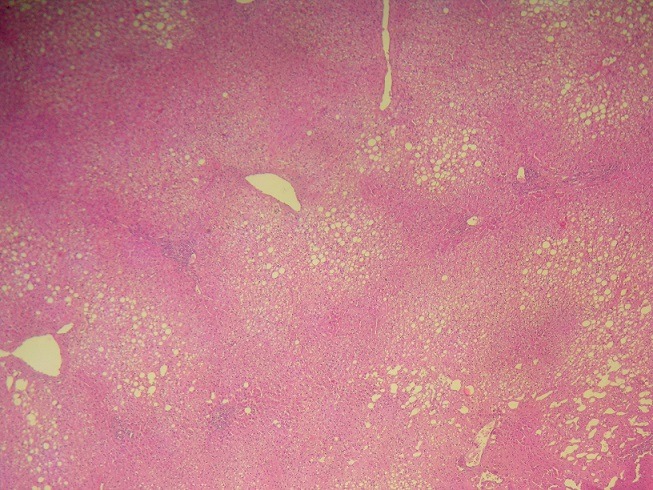
Macrovacuolar steatosis involving approximately 30% of the hepatocytes (hematoxylin and eosin, magnification × 40)

**Figure 4:**
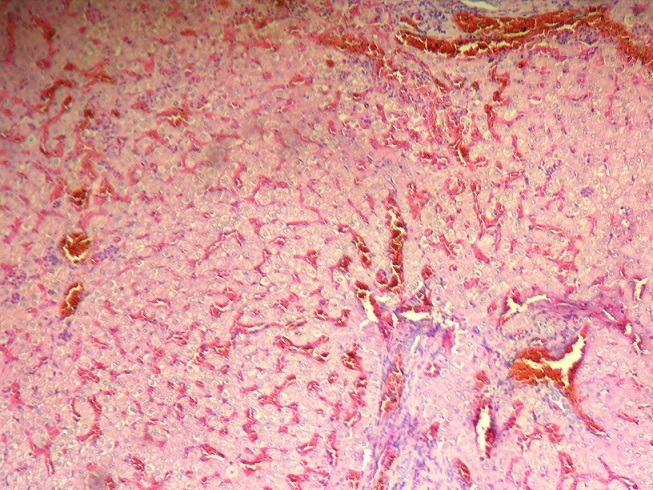
Sinusoidal obstruction syndrome characterized by distinct areas of dilated sinusoids with congestion (hematoxylin and eosin, magnification × 200)

**Description of histological lesions of the liver**: Histological examination of non-tumorous liver parenchyma demonstrated hepatic steatosis ≤ 30% in 15 cases (31%) (Figure 3) and steatosis >30% in 9 (19%) patients. No patients had NASH (median NAS score of 2 (range: 0-4)). One (2%), 12 (25%) and 10 (21%) patients had grade I, grade II, and grade III sinusoidal dilatation (Figure 4), respectively. Nodular regenerative hyperplasia (NRH) was observed in 3 (6%) patients. Perisinusoidal fibrosis was minimal in one (2%) patient and moderate in one (2%) patient. Forty-six patients had F0 fibrosis (96%) and two patients (4%) had F1 portal fibrosis. “Surgical hepatitis” lesions were described in 5 (10%) cases.

**Follow-up and evolution**: The mean follow-up period of our patients was 36 months. Four patients died after a mean follow-up period of 15 months. Local recurrence of the tumor occurred in three cases and only one patient had pulmonary metastases six months postoperatively. The other patients are still being followed-up.

## Discussion

The most commonly used CLM chemotherapy regimens include oxaliplatin (OX) plus 5-fluorouracil (5-FU) and leucovorin (FOLFOX) and irinotecan plus 5-FU and leucovorin (FOLFIRI). As chemotherapy is often administered prior to hepatic resection, adverse effects on the background liver parenchyma of CLM patients are increasingly recognized [4-6]. The importance of hepatic steatosis in patients undergoing liver resection was demonstrated in a meta-analysis by de *Meijer* et al, which showed its presence to be a risk factor for increased perioperative morbidity and mortality in patients undergoing major hepatic resection ((three Couinaud segments). In patients with steatosis > 30% three Couinaud segments). In patients with steatosis > 30%, the risk of death after major resection increased nearly threefold [7]. The severity of hepatic steatosis is determined by the proportion of involved hepatocytes as judged by histological review of hematoxylin and eosin-stained sections of the liver. A variety of grading systems exist, although the most commonly used is that proposed by *Kleiner* et al, which classifies steatosis as absent ( < 5% hepatocytes), mild (5-33% hepatocytes), moderate (> 33-66% hepatocytes) and severe (> 66% of hepatocytes) [8]. However, the association between chemotherapy and steatosis/steatohepatitis has not been universally accepted, and there is continuing debate over the relative importance of the number of chemotherapy cycles, their delivery, and patients individual traits, such as elevated body mass index [9, 10]. In our series, histological examination of non-tumorous liver parenchyma demonstrated steatosis ≤ 30% in 15 cases (31%) and steatosis > 30% in 9 (19%) patients. The presence of steatohepatitis is more worrying than simple steatosis when undertaking major liver resection and its presence has been demonstrated to be associated with increased surgical morbidity and mortality after resection of CLM [11].

In our series, there were no cases of steatohepatitis. Sinusoidal obstruction syndrome (SOS), previously termed veno-occlusive disease, is considered the result of severe toxic injury affecting hepatic sinusoidal endothelial cells. Sinusoidal obstruction syndrome has been associated with the use of OX-based systemic chemotherapy. Macroscopically, the affected liver has a characteristic bluish-red marbled appearance. Histologically, SOS is characterized by distinct areas of dilated sinusoids with congestion, which may be associated with liver cell plate atrophy. In severe cases, it can also be associated with perisinusoidal fibrosis, NRH, obstruction of centrilobular veins and peliotic change [12]. In our series, there were 23 cases of sinusoidal dilatation including grade I SOS (n = 1), grade II SOS (n = 12) and grade III SOS (n = 10). Perisinusoidal fibrosis was minimal in one (2%) case and moderate in one (2%) case. Recent meta-analysis studies have demonstrated that the nature of preoperative chemotherapy liver injury is a regimen-specific phenomenon [13]. For example, OX-based regimens are associated with sinusoidal injury, whereas irinotecan-based regimens are associated with steatohepatitis. In the context of liver surgery, chemotherapy-induced liver injury could increase the risks of intra-and postoperative complications and postoperative liver insufficiency [14]. Sinusoidal obstruction syndrome may also compromise liver regeneration in patients undergoing hepatectomy [15]. Therefore, both pathologists and clinicians should be aware of this syndrome as it has a relatively high prevalence and may affect patient outcomes. Nodular regenerative hyperplasia, a diffuse parenchymal reactive process, has been reported in 15% of patients receiving preoperative 5-FU-based chemotherapy for metastatic colorectal metastases [16]. Nodular regenerative hyperplasia is characterized by an ill-defined parenchymal nodularity formed by alternating areas of expanding nodules of hyperplastic hepatocytes with sinusoidal congestion and compression of peripheral parenchyma and central veins. The pathogenesis of NRH, which appears to develop within weeks of the completion of chemotherapy, is unknown, although it is believed to be related to a modification of the intrahepatic blood flow. However, no morphologic vascular alteration has been reported in that setting [16]. In our series, nodular regenerative hyperplasia was observed in 3 (6%) patients.

## Conclusion

In conclusion, most chemotherapeutic agents used for the management of CLM are associated with various histological patterns of liver injury but the incidence and pathogenesis are not well established. Concerns regarding chemotherapy-associated hepatotoxicity may negatively impact the ability to offer potentially curative therapy or increase morbidity in some patients. Although previously the domain of the oncologist, it is becoming increasingly important that the surgeon is aware of the mechanism of action and hepatotoxicity of these agents in order to predict and anticipate potential problems when the patient comes to surgery [17].

### What is known about this topic

- Most chemotherapeutic agents used for the management of CLM are associated with various histological patterns of liver injury, but the incidence and pathogenesis are not well established;
- Concerns regarding chemotherapy-associated hepatotoxicity may negatively impact the ability to offer potentially curative therapy or increase morbidity in some patients.

### What this study adds

- This is the first Tunisian study that describes the chemotherapy-induced major changes in the hepatic parenchyma and their prognostic impact in a Tunisian population.

## Competing interests

The authors declare no conflict of interest.
